# Grip strength performance from 9431 participants of the GenoFit study: normative data and associated factors

**DOI:** 10.1007/s11357-021-00410-5

**Published:** 2021-07-02

**Authors:** Jedd Pratt, Giuseppe De Vito, Marco Narici, Ricardo Segurado, Jackie Dolan, Judith Conroy, Colin Boreham

**Affiliations:** 1 Institute for Sport and Health, University College Dublin, Dublin, Ireland; 2 Genuity Science, Dublin, Ireland; 3 Department of Biomedical Sciences, CIR-Myo Myology Centre, Neuromuscular Physiology Laboratory, University of Padova, Padua, Italy; 4 Centre for Support and Training in Analysis and Research, and School of Public Health, Physiotherapy and Sports Science, University College Dublin, Dublin, Ireland

**Keywords:** Muscle strength, Normative data, Sarcopenia, Screening, Diagnosis

## Abstract

Weak grip strength is a strong predictor of multiple adverse health outcomes and an integral diagnostic component of sarcopenia. However, the limited availability of normative data for certain populations impedes the interpretation of grip performance across adulthood. This study aimed to establish normative data and low grip strength thresholds in a large adult population, and to examine associations between grip strength and clinically relevant health variables. A total of 9431 adults aged between 18 and 92 years participated in this study (mean age: 44.8 ± 13.4 years; 57% females). Grip strength, body composition, and cardiorespiratory (CR) fitness were assessed using hand dynamometry, dual-energy x-ray absorptiometry and physical work capacity tests, respectively. Low grip strength was established according to criteria of the European Working Group on Sarcopenia in Older People. Normative data and t-scores, stratified by sex and age groups, are presented. Grip performance was associated with lean mass, skeletal muscle index (SMI), fat mass, CR fitness, bone mineral density (BMD), android/gynoid ratio, disease prevalence and physical activity levels (all *p* < 0.001) after controlling for multiple potential confounders. Individuals with weak grip strength had lower lean mass, SMI, CR fitness (all *p* < 0.001) and BMD (*p* = 0.001), and higher disease prevalence (*p* < 0.001), compared to healthy controls, although sex-specific differences were observed. Grip strength has practical screening utility across a range of health domains. The normative data and grip strength thresholds established in this study can guide the clinical interpretation of grip performance and facilitate timely therapeutic strategies targeting sarcopenia.

## Introduction

The age-associated deterioration in muscle strength, mass and function, known as ‘sarcopenia’, is a fundamental contributor to the loss of independence among the elderly [1, 2]. In particular, low muscle strength has been identified as an independent risk factor for all-cause mortality, irrespective of muscle mass [3, 4]. While broadly stable during early adulthood (20–39 years), a progressive decline in muscle strength commences as early as at ~ 45 years of age and accelerates in later life [5, 6]. Such rapid deterioration, coupled with the plethora of unfavourable correlates of low muscle strength [3, 4, 7–9], emphasises the importance of timely diagnostic and therapeutic protocols. Moreover, because muscle strength in late adulthood is greatly affected by the peak attained during early adulthood [10], screening and developing muscle strength in young adults should be a high priority.

Grip strength assessment is well-established as a cost-effective, accessible means of muscle strength determination [11]. Weak grip strength is strongly associated with an array of clinical outcomes including overall morbidity [12] and mortality [13, 14], and is an integral diagnostic component of sarcopenia [15]. Moreover, the simplicity and reliability of grip strength determination underscore its potential utility as a screening tool [11]. Importantly, however, such diagnostic utility is dependent on the availability of normative data from relevant populations. Indeed, the use of reference values from the target population is fundamental for the accurate interpretation of grip performance [15]. While many studies have established such normative values for older adults [16–20], there are fewer data available for middle-aged adult and young adult populations. Consequently, the potential to identify those with, or at risk of, low grip strength is greatly limited for these cohorts.

In addition to the provision of normative data, the importance of the diagnostic value of grip strength has gained credence. While evidence to support associations across a spectrum of health and demographic variables is undoubtedly present [17, 18, 21–23], there are certain limitations to existing research. For example, although evidence of the association between grip strength, anthropometric and sociodemographic variables is common [17, 18, 20, 24], research supporting associations of grip strength with domains such as muscle mass, bone mineral density (BMD) and cardiorespiratory (CR) fitness, which may be more clinically relevant, is less abundant. Moreover, the availability of such evidence from large, age-diverse samples is even more sparse. Bearing this in mind, there is a clear need to examine the relationship between grip strength and multiple, clinically relevant health variables in large samples which are more representative of the general population. Such exploration, coupled with the generation of normative data, will improve clinical practice by enhancing the screening utility of grip strength and facilitating a more rapid implementation of preservative measures for skeletal muscle.

Accordingly, the primary aim of this study was to establish normative data and low grip strength thresholds from a large population from young adulthood to old age. A secondary aim was to assess the clinical utility of grip strength for health status screening.

## Methods

### Participant characteristics

Participants were recruited between September 2017 and October 2020 as part of the GenoFit study, a large-scale cross-sectional study taking place across two clinics in Ireland aiming to explore the impact of genetics, fitness and lifestyle on health. Nine-thousand four-hundred and thirty-one participants aged between 18 and 92 years took part in the current study (4051 males and 5380 females). To be eligible, participants had to be aged ≥ 18 years, be free from any severe cognitive disorder and/or musculoskeletal impairment that may affect muscle mass and/or muscle strength (e.g. injury to the hand, wrist or arm, or peripheral neuropathies such as carpal tunnel syndrome) and be willing to provide informed consent. The study protocol was approved by the Human Research Ethics Committee, University College Dublin. Written informed consent and a Physical Activity Readiness Questionnaire were obtained from all participants at enrolment.

### Anthropometry

A SECA stadiometer (SECA, Hamburg, Germany) and weighing scales were used to determine height and body mass, respectively, with participants dressed lightly and without shoes. Body mass index (BMI) was determined as body mass divided by height squared (kg/m^2^).

### Grip strength testing

Grip strength was assessed by trained study personnel using a Jamar digital hand-held dynamometer (JLW Instruments, Chicago, IL, USA) according to a previously described protocol [25]. In a standing position, subjects completed two maximal attempts (≥ 3 s) with each hand with their arm positioned straight by their side. The dynamometer was adjusted so that the middle phalanx was at ~ 90° to the handle. The average of the highest score from each hand was included in the analysis.

### Body composition and bone mineral density analysis

Dual-energy X-ray absorptiometry (DEXA) (Lunar Prodigy, GE Healthcare Technologies, USA) was used to determine body composition and BMD. Android to gynoid (A/G) ratio was determined as android fat mass divided by gynoid fat mass, while total BMD was used for analysis. Appendicular lean mass (ALM) was calculated as the combined lean mass of the limbs. Skeletal muscle index (SMI) was determined as ALM divided by height squared (kg/m^2^). A registered physician referred all DEXA scans while a trained technician performed the scans.

### Cardiorespiratory fitness testing

Cardiorespiratory fitness, measured as maximal oxygen uptake (VO_2max_), was predicted by a Physical Work Capacity test. The testing protocol was based on well-established World Health Organization guidelines [26]. Briefly, the 15-min test was performed on a cycle ergometer and consisted of a short warm-up followed by 3- and 4-min stages. Guided by trained personnel, participants maintained a steady cadence (60 rpm) throughout the test, while the load (watts) was increased at the beginning of each stage. Load increments were selected in order to achieve 55%, 65% and 75% of the participant’s maximum predicted heart rate (220—age) in the first, second and third stages, respectively. Heart rate was monitored throughout the test using a Polar heart rate monitor (Polar CIC, USA) and recorded in the last 30 s of each stage. After the test, heart rate, load, age and weight data were used to extrapolate predicted VO_2max_ using the following formula [27]:

$${\text{Wmax}} = {\text{load}}3\left( {\text{W}} \right) + \left[ {\left( {{\text{HRmax}} - {\text{HR}}3} \right) \times \frac{{{\text{load}}3\left( {\text{W}} \right) - \frac{{{\text{load}}1\left( {\text{W}} \right) + {\text{load}}2\left( {\text{W}} \right)}}{2}}}{{{\text{HR}}3 - \frac{{{\text{HR}}1 + {\text{HR}}2}}{2}}}} \right]$$

$${\text{VO}}2\max = \frac{{{\text{Wmax}} \times 12.48 + 217}}{{{\text{weight}}}}$$

### Health and lifestyle questionnaire

A self-reported questionnaire assessed the prevalence of diseases/disorders, medication intake, and level of physical activity and education. The prevalence of 56 diseases/disorders such as heart diseases/disorders, skin disorders, digestive and bowel disorders, breathing disorders and diabetes was assessed by the following question: “Have you received a medical diagnosis from a doctor for any of the following conditions? Please select all that apply”. Medication intake was assessed by asking: “Please list any medications you are currently taking (including antibiotics, oral/implant contraceptive or hormone replacement therapy)”. Physical activity levels were determined by asking: “How many days per week do you do at least 30 min of exercise that increases your breathing and heart-rate (e.g. brisk walking, jogging, cycling, swimming)?” Finally, level of education was determined by asking: “What is the highest level of education you have completed to date (no formal education, primary, lower secondary, higher secondary, third level or postgraduate)?”.

### Statistical analysis

Results are displayed as mean ± standard deviation (SD), unless stated otherwise. The data were tested for normality using residual plots, and skewness and kurtosis data. All data were normally distributed. Independent sample Student’s t-tests were used to assess differences between population characteristics according to gender. Spearman’s correlation coefficient was used to investigate the association between grip strength, SMI and age, due to the non-linear relationships between these variables. Multiple linear regression models were used to examine the association between grip strength and each clinical health variable (lean mass, SMI, fat mass, CR fitness, BMD, A/G ratio, disease prevalence and physical activity). Adjustment was made in a model-specific manner for potential confounders such as sex, age, BMI, disease prevalence, and level of physical activity and education. Analysis of covariance (ANCOVA) was performed to assess differences in means of clinical health variables between those with low and normal grip strength aged between 60 and 92 years, stratified by sex. Sex-specific percentile curves for grip strength and SMI were generated using the lambda‐mu‐sigma (LMS) method [28]. T-scores were derived for each participant’s grip strength by calculating the difference between the individual value and the mean of a young adult population, divided by the SD of the young adult population (20–39 years of age). Low grip strength thresholds were determined according to the criteria of the European Working Group on Sarcopenia in Older People (EWGSOP2) [15] as − 2 SDs below the sex-specific mean of a young adult population. All statistical analyses were performed using SPSS software (Version 26, IBM SPSS Inc., Chicago, IL, USA) and *p* < 0.05 was considered statistically significant for all tests.

## Results

### Study sample

The main characteristics of the study sample are illustrated in Table 1. A total of 9431 individuals aged between 18 and 92 years took part in this study (males, *n* = 4051; mean age: 42.5 ± 13.3 years; age range: 18–92 years, and females, *n* = 5380; mean age: 46.5 ± 13.1 years; age range: 18–87 years). None of the potential participants were unwilling to participate in the study. Level of education did not differ significantly between sexes, with most males (52.4%) and females (55.5%) having completed third-level education or above. Prevalence of disease among males was significantly lower compared to females (0.9 vs 1.3 diseases; *p* < 0.001). Males were also significantly more active than females (4.3 vs 4.0 days; *p* < 0.001). A total of 2141 participants were taking some form of medication (males, *n* = 773; females, *n* = 1368). Among the most common reasons for medicating were contraception (*n* = 272), hypertension (*n* = 235), asthma (*n* = 175), hypothyroidism (*n* = 153), hypercholesterolemia (*n* = 128) and depression/anxiety (*n* = 147).

**Table 1:** Participant characteristics stratified by sex

Parameter	Male (*n* = 4051)	Female (*n* = 5380)	Total (*n* = 9431)	P-value
Sociodemographic				
Age (years)	42.5 ± 13.3	46.5 ± 13.1	44.8 ± 13.4	< 0.001
Education, n (%)				
No formal education	4 (0.1)	1 (< 0.0)	5 (0.1)	0.124
Primary education	33 (0.8)	35 (0.6)	68 (0.7)	
Lower secondary	167 (4.1)	152 (2.8)	319 (3.3)	
Higher secondary	558 (13.8)	724 (13.5)	1282 (13.6)	
Third-level degree	2124 (52.4)	2984 (55.5)	5108 (54.2)	
Postgraduate degree	1165 (28.8)	1484 (27.6)	2649 (28.1)	
Anthropometric				
Height (cm)	178.9 ± 6.8	164.9 ± 6.3	170.9 ± 9.5	< 0.001
Body mass (kg)	83.6 ± 12	66.5 ± 10.8	73.8 ± 14.1	< 0.001
Body mass index (kg/m^2^)	26.1 ± 3.3	24.5 ± 3.8	25.2 ± 3.7	< 0.001
Body composition				
Lean mass (kg)	60.74 ± 7.4	42.19 ± 5.4	50.16 ± 11.1	< 0.001
Skeletal muscle index (kg/m^2^)	8.92 ± 1.0	6.77 ± 0.8	7.69 ± 1.4	< 0.001
Fat mass (kg)	20.51 ± 8.5	22.62 ± 8.2	21.72 ± 8.4	< 0.001
Bone mineral density (g/cm^2^)	1.34 ± 0.12	1.18 ± 0.11	1.25 ± 0.14	< 0.001
Android/gynoid ratio	0.58 ± 0.20	0.37 ± 0.16	0.46 ± 0.21	< 0.001
Strength and fitness				
Grip strength (kg)	49.3 ± 8.6	30.3 ± 5.4	38.5 ± 10.5	< 0.001
VO2_max_ (ml/kg/min)	43.9 ± 10.2	34.4 ± 8.9	38.5 ± 10.5	< 0.001
Health and lifestyle				
No. of diseases/disorders	0.9 ± 1.1	1.3 ± 1.4	1.1 ± 1.3	< 0.001
Physical activity	4.3 ± 2	4.0 ± 2.1	4.1 ± 2.1	< 0.001

Physical activity: days per week performing ≥ 30 min of moderate intensity exercise

### Normative grip strength data and t-scores

Detailed grip strength data according to age group and sex are presented in Table 2. Overall, males had significantly higher grip strength than females (49.3 kg and 30.3 kg, respectively; *p* < 0.001). Similarly, across the entire sample, males had higher t-scores compared to females (− 0.20 and − 0.33, respectively). For both sexes, grip strength performance was highest among the 30–39-year age group (mean = 51.3 kg for males and 32.3 kg for females) and lowest among the ≥ 80-year age group (mean = 34.4 kg for males and 20.6 kg for females).

**Table 2:** Normative grip strength data and t-scores stratified by sex and age

Age group	n	Grip strength (kg)								Average t-score^a^ (95% CI)
		Percentiles							Mean ± SD	
		5th	10th	25th	50th	75th	90th	95th		
Males										
18–29	730	37.0	39.3	44.4	50.4	55.8	62.0	65.2	50.5 ± 8.7	− 0.06 (− 0.14, 0.01)
30–39	1132	37.6	40.7	45.7	51.1	56.7	62.3	65.5	51.3 ± 8.5	0.03 (− 0.03, 0.09)
40–49	1018	38	40.7	45.4	50.1	55.8	60.8	65.0	50.6 ± 7.8	− 0.05 (− 0.11, 0.01)
50–59	669	36.1	38.3	42.6	48.0	53.5	57.4	60.4	47.9 ± 7.8	− 0.36 (− 0.43, − 0.29)
60–69	365	32.8	34.9	38.6	43.3	48.9	54.0	56.9	43.9 ± 7.5	− 0.83 (− 0.92, − 0.74)
70–79	120	30.0	31.3	33.9	38.7	42.8	46.3	49.3	38.9 ± 6.3	− 1.42 (− 1.56, − 1.29)
≥ 80	17^b^	-	-	-	-	-	-	-	34.4 ± 6.3	− 1.95 (− 2.33, − 1.57)
Total	4051	35.4	38.4	43.5	49.1	55.0	60.4	63.8	49.3 ± 8.6	− 0.20 (− 0.23, − 0.17)
Females										
18–29	658	23.8	25.0	28.2	31.5	35.0	38.8	40.7	31.7 ± 5.3	− 0.07 (− 0.15, 0.01)
30–39	1053	24.0	25.8	28.7	32.2	35.5	38.6	40.9	32.3 ± 5.2	0.04 (− 0.02, 0.10)
40–49	1353	23.7	25.6	28.5	31.7	35	38.3	40.4	31.8 ± 5.0	− 0.05 (− 0.10, 0.01)
50–59	1334	21.9	23.6	26.2	29.4	32.5	35.5	37.9	29.5 ± 4.9	− 0.50 (− 0.55, − 0.45)
60–69	811	19.9	21.6	24.0	26.8	29.9	32.8	34.4	27.0 ± 4.5	− 0.98 (− 1.04, − 0.92)
70–79	158	18.7	19.6	21.3	24.0	27.0	29.6	32.8	24.4 ± 4.1	− 1.48 (− 1.60, − 1.35)
≥ 80	13^b^	-	-	-	-	-	-	-	20.6 ± 5.4	− 2.21 (− 2.84, − 1.58)
Total	5380	21.8	23.6	26.7	30.2	33.8	37.2	39.4	30.3 ± 5.4	− 0.33 (− 0.36, − 0.31)

^a^t-scores determined as number of standard deviations away from the mean of a young reference population; ^b^percentile values not shown for ≥ 80 age groups due to limited data availability

**Fig. 1:**
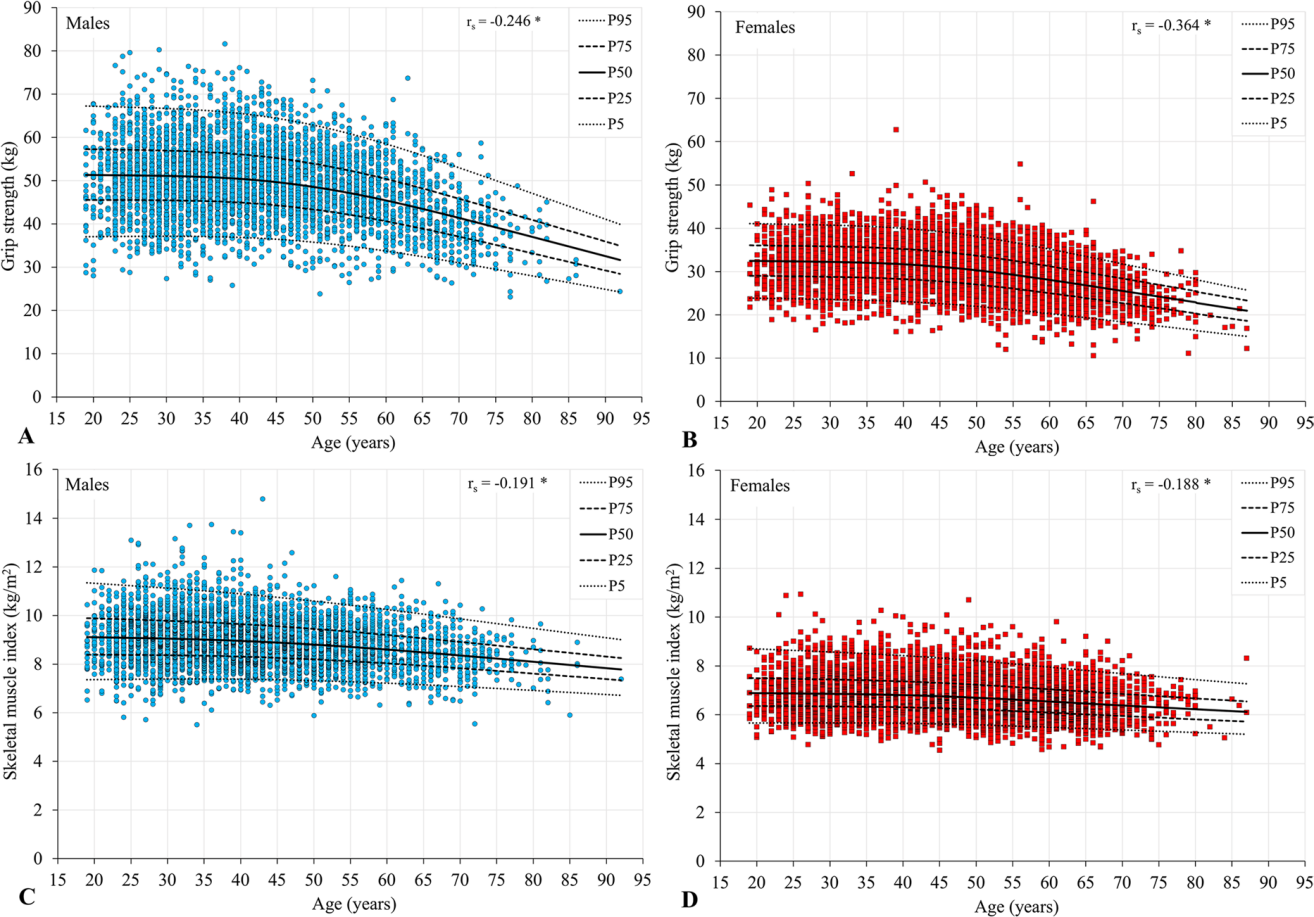
Association between grip strength and age (panels **A**, **B**) and skeletal muscle index and age (panels **C**, **D**) including percentile curves (**A**, **C** = males, *n* = 4051; **B**, **D** = females, *n* = 5380; *p < 0.001)

Figure 1 illustrates the association between grip strength, SMI and age for males and females. Significant negative correlations were observed between grip strength and age for both males and females (r_s_ =  − 0.246; *p* < 0.001 and r_s_ =  − 0.364; *p* < 0.001, respectively). For males, grip strength performance remained relatively stable until ~ 50 years of age; after which, the decline in grip strength accelerated. For females, the start of decline appeared to occur sooner, at ~ 45 years of age. Similarly, a more rapid deterioration in t-scores was observed in females after 50 years of age, compared to males. Interestingly, SMI remained more stable across adulthood than grip strength (r_s_ =  − 0.191; *p* < 0.001 and r_s_ =  − 0.188; *p* < 0.001, for males and females respectively), indicating a more rapid age-related decline in muscle strength than muscle mass.

### Low grip strength thresholds and prevalence

**Fig. 2:**
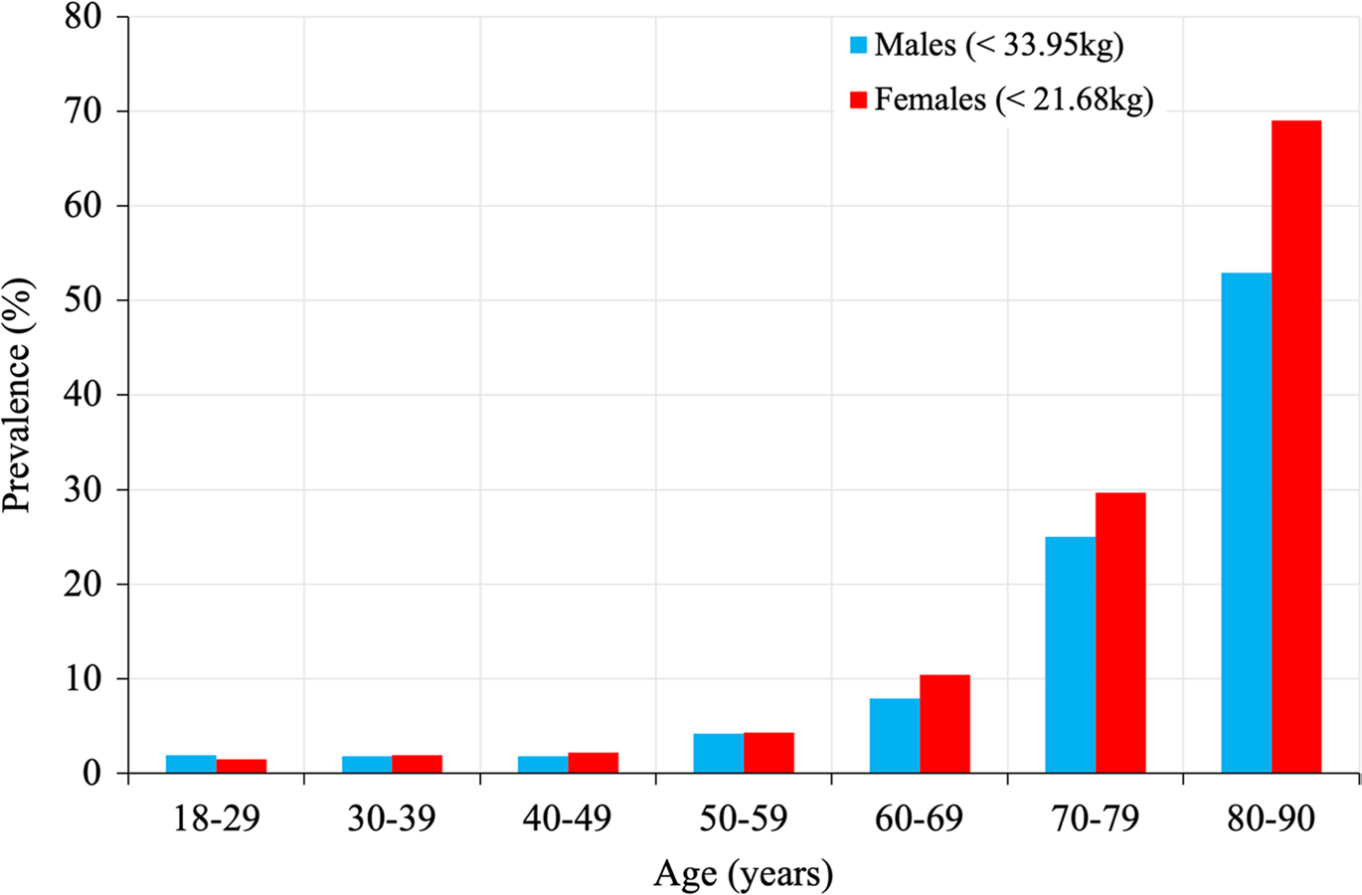
Estimated prevalence of low grip strength according to sex (low grip =  − 2 standard deviations below young adult mean)

Low grip strength thresholds of − 2 SDs below the young adult mean were determined as < 33.95 kg for males and < 21.68 kg for females (young adult population mean; males: *n* = 1842, mean = 51.03 kg, SD = 8.54 kg; females: *n* = 1705, mean = 32.06 kg, SD = 5.19 kg). Prevalence of low grip strength remained relatively stable for both sexes until ~ 60 years of age, after which there was a considerable increase (Fig. 2). In males, 7.9% of individuals aged 60–69 years had low grip strength, which increased substantially to 25% and 52.9% of those aged 70–79 and 80–89 years, respectively. Low grip strength became progressively prevalent among females, at 10.4%, 29.7% and 69% for those aged 60–69, 70–79 and 80–87 years, respectively.

### Factors associated with grip strength

Multiple regression models assessing the association between handgrip strength and each clinical health variable are presented in Table 3. Grip strength was significantly associated with lean mass (95% CI, 0.384 to 0.413), SMI (95% CI, 0.037 to 0.040), fat mass (95% CI, − 0.114 to − 0.087), BMD (95% CI, 0.003 to 0.004), A/G ratio (95% CI, − 0.003 to − 0.002) and disease/disorder prevalence (95% CI, − 0.017 to − 0.009) after controlling for a multitude of potential confounders (all *p* < 0.001). Interestingly, significant relationships were also observed between grip strength, level of physical activity (95% CI, 0.016 to 0.028) and CR fitness (95% CI, 0.128 to 0.175), after adjustment for sex, age, BMI, pathology and education (both *p* < 0.001).

**Table 3:** Multiple regression models assessing the association between grip strength and each clinical health variable

Variables	Unstandardised coefficients	95% CI	P-value	R^2^
Model 1^a^—Lean mass (kg)	0.398	0.384, 0.413	< 0.001	0.819
Model 2^a^—Skeletal muscle index (kg/m^2^)	0.039	0.037, 0.040	< 0.001	0.841
Model 3^a^—Fat mass (kg)	− 0.100	− 0.114, − 0.087	< 0.001	0.727
Model 4^a^—VO2_max_ (ml/kg/min)	0.151	0.128, 0.175	< 0.001	0.504
Model 5^a^—Bone mineral density (g/cm^2^)	0.004	0.003, 0.004	< 0.001	0.475
Model 6^a^—Android/gynoid ratio	− 0.002	− 0.003, − 0.002	< 0.001	0.614
Model 7^b^—Disease prevalence	− 0.013	− 0.017, − 0.009	< 0.001	0.092
Model 8^c^—Physical activity	0.022	0.016, 0.028	< 0.001	0.037

*n* = 9431; independent variable for all models = grip strength; adjustments: ^a^sex, age, body mass index (BMI), disease prevalence, and level of physical activity and education, ^b^sex, age, BMI, and level of physical activity and education, ^c^sex, age, BMI, disease prevalence and level of education

ANCOVA revealed that in individuals aged between 60 and 92 years, those with low grip strength had significantly lower lean mass (42.63 vs 46.03 kg, *p* < 0.001), SMI (6.85 vs 7.20 kg/m^2^, *p* < 0.001), CR fitness (28.01 vs 31.04 ml/kg/min, *p* < 0.001) and BMD (1.12 vs 1.16 g/cm^2^, *p* = 0.001) compared to healthy controls (Table 4). These associations remained significant after controlling for age, BMI, disease prevalence, and level of physical activity and education (Table 4). Additionally, disease prevalence was significantly higher in those with low grip strength (2.17 vs 1.70 diseases, *p* < 0.001), when compared with those with normal grip strength. In males, individuals with low grip strength had significantly lower lean mass (54.93 vs 57.01 kg, *p* = 0.007), SMI (8.23 vs 8.49 kg/m^2^, *p* = 0.003) and CR fitness (32.77 vs 35.45 ml/kg/min, *p* = 0.014), after adjusting for the same covariates. A trend was also observed for a higher disease prevalence in males with low grip strength (1.76 vs 1.51 diseases, *p* = 0.197), compared to healthy controls. In the female population, those with weak grip strength had significantly lower lean mass (37.40 vs 40.25 kg, *p* < 0.001), SMI (6.26 vs 6.52 kg/m^2^, *p* < 0.001), BMD (1.06 vs 1.09 g/cm^2^, *p* = 0.003) and physical activity levels (3.69 vs 4.15 days, *p* = 0.028), after adjusting for the relevant covariates. Moreover, disease prevalence was significantly higher among females with low grip strength (2.17 vs 1.83 diseases, *p* = 0.022) than those with normal grip strength. While not statistically significant, lower CR fitness was also observed in females with weak grip strength (27.80 vs 28.43 ml/kg/min, *p* = 0.290).

**Table 4:** Unadjusted and adjusted associations between grip strength and clinical variables in those aged between 60 and 92 years

Parameter	Low grip (*n* = 208)	Normal grip (*n* = 1276)	P-value	Low grip (*n* = 208)	Normal grip (*n* = 1276)	P-value
	Unadjusted means (SEM)			Adjusted means (SEM)		
Lean mass (kg)^a^						
All subjects	42.63 (0.64)	46.03 (0.27)	< 0.001	42.61 (0.62)	46.03 (0.24)	< 0.001
Males	54.05 (0.75)	57.15 (0.31)	< 0.001	54.93 (0.70)	57.01 (0.26)	0.007
Females	37.08 (0.31)	40.30 (0.16)	< 0.001	37.40 (0.37)	40.25 (0.15)	< 0.001
Skeletal muscle index (kg/m^2^)^a^						
All subjects	6.85 (0.08)	7.20 (0.03)	< 0.001	6.85 (0.07)	7.20 (0.03)	< 0.001
Males	8.20 (0.11)	8.49 (0.04)	0.008	8.23 (0.08)	8.49 (0.03)	0.003
Females	6.19 (0.06)	6.53 (0.02)	< 0.001	6.26 (0.04)	6.52 (0.02)	< 0.001
VO2_max_ (ml/kg/min)^a^						
All subjects	28.01 (0.51)	31.04 (0.23)	< 0.001	29.00 (0.56)	30.88 (0.22)	0.002
Males	30.33 (0.88)	35.83 (0.42)	< 0.001	32.77 (1.00)	35.45 (0.38)	0.014
Females	26.89 (0.60)	28.58 (0.24)	0.007	27.80 (0.54)	28.43 (0.22)	0.290
Bone density (g/cm^2^)^a^						
All subjects	1.12 (0.01)	1.16 (0.01)	0.001	1.13 (0.01)	1.16 (< 0.00)	0.004
Males	1.29 (0.01)	1.30 (0.01)	0.270	1.30 (0.02)	1.30 (0.01)	0.874
Females	1.05 (0.01)	1.09 (< 0.00)	< 0.001	1.06 (0.01)	1.09 (< 0.00)	0.003
No. of diseases/disorders^b^						
All subjects	2.17 (0.12)	1.70 (0.04)	< 0.001	2.05 (0.11)	1.72 (0.04)	0.005
Males	1.85 (0.18)	1.49 (0.07)	0.046	1.76 (0.18)	1.51 (0.07)	0.197
Females	2.32 (0.16)	1.81 (0.05)	< 0.001	2.17 (0.14)	1.83 (0.05)	0.022
Physical activity^c^						
All subjects	3.94 (0.17)	4.11 (0.06)	0.317	3.91 (0.16)	4.11 (0.06)	0.238
Males	4.29 (0.30)	4.06 (0.10)	0.411	4.32 (0.28)	4.06 (0.11)	0.394
Females	3.77 (0.20)	4.13 (0.08)	0.075	3.69 (0.19)	4.15 (0.08)	0.028

Low grip =  − 2 standard deviations below young adult mean; adjustments: ^a^age, body mass index (BMI), disease prevalence, and level of physical activity and education, ^b^age, BMI, and level of physical activity and education, ^c^age, BMI, disease prevalence and level of education. *SEM*, standard error of mean

## Discussion

### Main findings

This study presents normative data and low grip strength thresholds established from a very large sample (*n* = 9431) of individuals aged between 18 and 92 years with well-characterised phenotypic data. Grip strength was strongly associated with lean mass, SMI, fat mass, CR fitness, BMD, A/G ratio, disease prevalence and physical activity levels (all *p* < 0.001). Individuals with low grip strength had significantly poorer outcomes across multiple clinically relevant health domains such as lean mass, SMI, CR fitness, disease prevalence (all *p* < 0.001) and BMD (*p* = 0.001), compared to those with normal grip strength. A progressive deterioration in grip performance was observed from 45 years of age.

### Grip performance across adulthood

Our findings show that for both sexes, grip strength performance remains relatively stable during early adulthood (18–39 years), peaks between 30 and 39 years of age (male peak = 51.3 kg; female peak = 32.2 kg) and then stabilises for a brief period before deteriorating. In males, a progressive degradation of grip performance was observed from ~ 50 years of age, while the corresponding decline occurred sooner in females, at ~ 45 years (Fig. 1). For both sexes, this deterioration accelerated after 60 years of age, and further accelerated after 80 years. These findings are in accordance with previous reports [6, 29, 30], illustrating the presence of three main phases of grip strength across adulthood: (1) slight increase during early adulthood, peaking between 30 and 39 years; (2) maintenance during midlife (40–45/50 years); (3) progressive deterioration from late middle age (≥ 45/50 years), accelerating after 60 years of age. Importantly, our study confirms the presence of a progressive decline in grip strength commencing as early as ~ 45 years of age. This observation is of particular relevance as it suggests the need for early screening and therapeutic interventions targeting skeletal muscle preservation. The implementation of such measures during early adulthood may be particularly beneficial in attenuating the rate of grip strength decline observed during late adulthood.

Interestingly, a study from 1991 reported such decline in grip strength to occur from 40 and 30 years of age in males and females, respectively [31], notably sooner than observed in our study and other more recent studies [6, 29, 30]. Such differences are possibly due to developments in education, healthcare services and public health awareness in recent decades. Therefore, while our study confirms the presence of an early degradation of grip strength, the progress in delaying the onset of such decline should encourage future endeavours to further attenuate the age-associated deterioration in skeletal muscle health.

### Comparisons with previous studies

While many studies have examined grip performance within older European populations, there remains a paucity of grip strength data across the lifespan. To date, only two studies have provided primary data, in Swiss [32] and Italian [5] populations, while two recent pooled analysis studies have also provided life course data for British [29] and German [6] populations. When our results from each stage of adulthood (early = 18–39 years, middle = 40–59 years and late ≥ 60 years) were compared with those from other studies, our findings were either slightly lower (~ 6%) [6, 32], slightly higher (~ 6%) [29], or notably higher (~ 14%) [5] than those of previous reports. Importantly, however, small sample sizes [32] and heterogeneous gender distributions, sample sizes and grip strength determination protocols [29] limit the quality of data in some cases and make direct comparisons difficult. Our study also provides, for the first time, national data across the adult age range for an Irish population, the only previous study providing grip strength results for those only aged ≥ 50 years [19].

### Clinical interpretation of grip strength

Low grip strength thresholds derived from an appropriate reference population are fundamental for the clinical interpretation of grip strength performance. The present study provides clinically relevant cutoff points for grip strength for a large population spanning the entire adult age span. In accordance with EWGSOP2 criteria, low grip strength was defined as < 33.95 kg and < 21.68 kg for males and females, respectively. At this cutoff, prevalence of low grip strength was high, with 52.9% of males and 69% of females aged ≥ 80 years having weak grip strength. While such thresholds are relatively high, they are similar to other recent studies that used a similar − 2 SD approach within European populations (< 32 kg and < 33 kg, < 19 kg and < 21 kg, for males and females, respectively) [6, 29]. Moreover, although the thresholds established in our study are notably higher than those proposed by the EWGSOP2 for sarcopenia diagnosis (27 kg and 16 kg for males and females, respectively), they are similar to those recently suggested by the Sarcopenia Definitions and Outcomes Consortium (SDOC) (35.5 kg and 20 kg for males and females, respectively) [33]. Intriguingly, the SDOC cutoff thresholds have since been shown to demonstrate strong prognostic power across an array of clinical outcomes such as falls, hip fracture, mobility limitations and death [34]. Furthermore, as will be discussed, our findings support the screening utility of higher cutoff points, across a suite of relevant health domains. With that in mind, a higher threshold, as proposed in our study, may be more efficacious in isolating those at risk of clinical outcomes than those by the EWGSOP. Indeed, utilising grip strength thresholds as low as the EWGSOP suggests may risk classifying an individual’s muscle strength as ‘normal’, despite that person being at a higher risk for the aforementioned clinical outcomes.

In addition to diagnostic thresholds, the t-scores presented in Table 2 provide further clinical relevance in assessing grip strength across the complete performance spectrum. Together, our findings have clear translational potential in facilitating the identification of those with, or at risk of, low grip strength, and enabling the clinical interpretation of grip strength outside of distinct performance thresholds.

### Screening utility of grip strength

In addition to generating useful reference data for handgrip across the adult lifespan, our study indicates the utility of grip strength as a health status screening tool. Importantly, we found grip performance to be significantly associated with SMI, after adjustment for multiple potential confounders. This finding, coupled with the more rapid age-related decline in grip strength compared to SMI (Fig. 1), reinforces the importance of timely screening and therapeutic protocols targeting muscle strength.

Additionally, we confirm grip strength to be significantly associated with lean mass, fat mass, CR fitness, BMD, A/G ratio, disease prevalence and physical activity levels. Such findings are consistent with previous studies, reporting associations between grip strength and lean mass [35], body fat % [35], BMD [36, 37], peak oxygen uptake (VO2_peak_) [38] and morbidity [12]. While no previous research has explored the relationship between grip performance and A/G ratio, studies have demonstrated significant negative associations between grip strength and waist circumference [17, 18]. Together, our findings complement existing research, further supporting the use of grip strength as a screening tool for overall health. However, importantly, the practicality of grip strength as a diagnostic tool is dependent on the availability of grip strength thresholds for specific populations.

In this regard, we assessed the association between thresholds for weak grip strength and various clinically relevant outcomes for those aged ≥ 60 years. Interestingly, even at this relatively high cutoff point (< 33.95 kg and < 21.68 kg for males and females, respectively), weak grip strength was associated with significantly poorer outcomes across an array of relevant health domains such as lean mass, SMI, BMD, CR fitness and disease prevalence, compared to those with normal grip strength.

However, the relevance of low grip strength as a screening tool is likely to be sex-specific. Our findings suggest that low grip performance may have greater clinical relevance among females than males, particularly when screening for BMD. Compared to those females with normal grip strength, those with low grip strength had significantly lower lean mass, SMI, BMD and levels of physical activity and significantly higher disease prevalence, while associations in the corresponding males were confined to lower lean mass, SMI and VO2 max. These findings are consistent with previous reports demonstrating stronger associations between low grip strength, BMD and physical activity levels among females, compared to males [39, 40]. While we found associations between low grip strength and disease prevalence in males only, and similar associations with CR fitness in females only, two recent studies have reported significant associations between these variables across both sexes [38, 41]. Such inconsistencies are underscored by an overall lack of research relating to specific grip strength cutoff points and health. Nonetheless, there are several plausible hypotheses underpinning the sex-specific differences observed in this study. Firstly, the clinical relevance of grip strength may increase at a more advanced stage of degradation, explaining the stronger associations observed in females, for whom strength levels are inherently lower. Secondly, the greater quantities of lean mass innately present in males may provide a stronger contribution towards the examined health domains, potentially weakening grip strength associations. Lastly, sex-specific differences in hormones and hormonal regulation may also contribute towards the disparities. Evidently, future research is needed to further illuminate the clinical pertinence of grip strength thresholds in health screening, and to establish whether this relevance is mediated by sex.

### Physical activity, cardiorespiratory fitness and grip strength

Our findings illustrate a progressive decline in grip performance commencing as early as ~ 45 years of age and a spectrum of unfavourable health correlates with weak grip strength. Accordingly, ascertaining measures to develop and preserve grip strength is a high priority. In that regard, physical activity and cardiorespiratory fitness are well-established contributors to successful aging, with preservative benefits across a range of health domains [42, 43]. Unsurprisingly therefore, we found significant associations between grip strength, physical activity levels and CR fitness. These findings are consistent with recent studies, reporting benefits of regular physical activity during midlife on follow-up grip performance in late adulthood [44], and a continuation of this association for those physically active into late adulthood [45, 46]. Interestingly, becoming physically sedentary has also been shown to induce a more rapid decline in grip strength [47], further emphasising the importance of maintaining an active lifestyle for preserving muscular performance.

While many studies have explored the relationship between physical activity and grip strength, there is limited evidence surrounding the importance of CR fitness on grip capabilities. Recently, however, CR fitness, measured as VO2_peak_, has been shown to be an independent predictor of grip strength among community dwellers [38]. Our results support such findings, demonstrating a significant relationship between grip strength and CR fitness, and highlighting that those with clinically low grip strength display significantly poorer CR performance. These findings are perhaps unsurprising considering the well-established relationship between muscle strength and lean mass [35], and the contribution of such metabolically active tissue towards CR performance [48]. Nonetheless, CR fitness is undoubtedly a multifaceted construct that encompasses factors such as muscle capillarisation, oxidative capacity and pulmonary function [49]. Therefore, more research is needed to establish the causal relationship between grip strength and CR performance.

While evidence supporting the benefits of physical activity and CR fitness on grip strength is undoubtedly present, more research is needed to establish whether the effects are mediated by sex. Indeed, our findings suggest the association between CR fitness, physical activity and grip strength may be particularly robust among females. Such postulation is in accordance with existing research, illustrating stronger associations between these domains in females compared with males [39, 46]. Nonetheless, evidence supporting the role of physical activity and CR fitness in enhancing grip strength in both sexes is also present [38, 44, 45]. In that regard, while future exploration of sex-specific relevance is needed, physical activity and CR training should be promoted as accessible means of enhancing and preserving grip strength across the lifespan. More specifically, encouraging regular physical activity during early adulthood may be particularly useful in attenuating early degradation in muscle strength.

### Strengths and limitations

The main strengths of this study are the large sample size and broad range of age groups (18–92 years) included. Moreover, unlike other studies [6, 29, 30], we gathered primary data to establish normative values. Additionally, the data were collected at only two sites, further enhancing data integrity. However, several limitations to this study should be acknowledged. Firstly, the suite of health and fitness assessments included in the GenoFit study protocol may have attracted a relatively healthy population. Nonetheless, the educational profile of our study sample was broadly similar, albeit slightly higher, to national educational attainment estimates from the Central Statistics Office (51% of those aged 25–64 years have at least third-level education) [50]. Secondly, the normative data in our study were gathered cross-sectionally; therefore, the percentile curves should not be used to monitor the course of an individual’s grip strength over time. Also, the cross-sectional design prevents the determination of causal relationships between variables. Furthermore, participant’s CR outputs were indirectly predicted, which may affect the accuracy of results. Nonetheless, the validity of such results was enhanced by the implementation of standardised protocols by experienced technicians. It is also important to note that 208 individuals (14%) aged ≥ 60 years had hand osteoarthritis, a limiting factor for grip strength. Furthermore, as with all self-reported data collection, inaccurate reporting is a possibility. As a result, there may be inaccuracies in such data gathered in this study, although the comprehensibility of questions coupled with the large sample size should help minimise such inconsistencies. Finally, future research may benefit from assessing ethnicity of participants.

## Conclusions and future direction

In summary, our study presents normative data and grip strength thresholds which may guide the clinical interpretation of grip performance and help identify those with, or at risk of low grip strength. Our findings illustrate a progressive decline in grip strength from ~ 45 years of age, emphasising the need for preventive therapeutic protocols. In that regard, increasing levels of physical activity and CR fitness should be encouraged as a means of enhancing and preserving grip strength. Novel insights are provided into the practical utility of grip strength as a screening tool for multiple, clinically relevant health domains. Nonetheless, further exploration is needed to establish sex-specific differences in the screening utility of grip strength and in the causative pathways between physical activity, CR fitness and grip performance.
